# Assessment of the Public’s Knowledge, Attitudes, and Practices Regarding Rabies in the Republic of Guinea

**DOI:** 10.1155/vmi/2932607

**Published:** 2026-10-06

**Authors:** Noumouké Kante, Abdoulaye Kuan Traore, Lanceï Kaba, Almamy Ousmane Deen Camara, Yacouba Konate, Cathérine Beimy, Pathé Navabb Kowane, Pépé Koly Guilavogui, Michel Alexis Sagno, Moussa Camara, Karim Tuo, Mohamed Magassouba, Kouramoudou Berete, Mohamed Idriss Doumbouya, Pépé Gbamou, Sayon Yombouno, Fassou Kourouma, Madou Sanou, Nafissatou Ouedraogo, Noël Tordo, Nicolas Barro

**Affiliations:** ^1^ Dalaba Higher Institute of Veterinary Sciences and Medicine Dalaba Guinea; ^2^ Norbert ZONGO University Koudougou Burkina Faso unz.bf; ^3^ Pasteur Institute of Côte d’Ivoire Abidjan Côte d’Ivoire pasteur.ci; ^4^ Central Veterinary Diagnostic Laboratory of Conakry Conakry Guinea; ^5^ National Directorate of Veterinary Services Conakry Guinea; ^6^ Joseph KI-ZERBO University Ouagadougou Burkina Faso; ^7^ Daniel Ouezzin Coulibaly University Ouagadougou Burkina Faso; ^8^ Pasteur Institute of Guinea Conakry Guinea

**Keywords:** attitudes and practices, cross-sectional study, Guinea, knowledge, public health, rabies, zoonosis

## Abstract

Rabies remains a major public health problem in resource‐limited countries, particularly in sub‐Saharan Africa. The objective of this study was to assess the knowledge, attitudes, and practices (KAP) of the population regarding rabies in the Republic of Guinea. A cross‐sectional study was conducted among 2733 participants in five regions (Boké, Kindia, Labé, Siguiri, and N’Zérékoré). Data were collected using a structured questionnaire administered via KoboCollect and then analyzed using DATAtab and R software. Descriptive analyses, chi‐square tests, and multivariable logistic regression analyses were used to identify associated factors. In total, 66% of respondents had heard of rabies, but significant gaps were observed regarding modes of transmission and prevention measures. Dog bites were identified as the primary mode of transmission by 37.87% of participants. Canine vaccination was recognized as a preventive measure by 45.01% of respondents. Attitudes were predominantly poor (88.84% scored below the predefined 70% acceptability threshold, i.e., “low/unfavorable attitude,” versus 10.79% moderate and 0.37% good), and 73.80% of participants exhibited inappropriate practices (versus 8.05% average and 18.15% good). Overall, 53.1% of respondents had a low level of knowledge, 29.3% an average level, and 17.6% a good level. A statistically significant association was observed between knowledge level and postexposure practices (*p* < 0.001). In a first multivariable logistic regression model (dependent variable: acceptable attitude, yes/no), educational level, age, and occupation were significantly associated with attitudes. In a second, separate multivariable model (dependent variable: dog bite, yes/no), none of the variables examined were significantly associated with dog bites, except age ≥ 50 years (adjusted OR = 1.98; 95% CI: 1.20–3.25; *p* = 0.006). These results highlight significant gaps in knowledge and practices related to rabies in Guinea. Strengthening targeted awareness campaigns, improving access to postexposure prophylaxis, and promoting canine vaccination are essential to reducing the burden of rabies. Because this study did not include animal, environmental, or surveillance data, an integrated “One Health” approach is recommended here as a forward‐looking direction for future multisectoral intervention, rather than a conclusion directly supported by the present findings.

## 1. Introduction

Rabies is an acute viral zoonosis that remains one of the most feared infectious diseases in public health due to its almost always fatal outcome once clinical signs appear. It is caused by a virus of the genus *Lyssavirus* and is transmitted to humans primarily through the saliva of an infected animal, most often following a bite [1, 2]. Despite the existence of effective preventive measures, the disease continues to pose a major public health problem in many regions of the world.

Globally, rabies is responsible for approximately 59,000 human deaths per year, with a high concentration in Africa and Asia [3]. This mortality persists despite the availability of postexposure prophylaxis (PEP), which benefits millions of people each year [4]. This situation is largely due to difficulties in accessing care, the high cost of vaccines, and a lack of information among exposed populations [5, 6].

In developing countries, dogs are the primary reservoir and vector for the transmission of rabies to humans [7]. The circulation of the virus is facilitated by the large number of stray dogs, low canine vaccination coverage, and the lack of effective control programs [8]. Furthermore, socioeconomic and cultural factors strongly influence the behaviors of at‐risk populations, particularly regarding seeking medical care after a bite.

Knowledge, attitudes, and practices (KAP) surveys are widely used to assess the level of public awareness and behavior regarding communicable diseases. Several studies have shown that, despite general knowledge of rabies, significant gaps persist regarding modes of transmission and preventive measures [9, 10]. More recent evidence confirms that this pattern persists: A systematic review of KAP studies in Ethiopia found that knowledge gaps and unfavorable attitudes remain widespread despite decades of awareness efforts [11], and household surveys published since 2023 continue to document limited knowledge of appropriate postexposure behavior even where general awareness of the disease is high [12, 13]. Furthermore, misconceptions, particularly regarding the possibility of treatment after symptoms appear, continue to be observed in several settings [14].

In sub‐Saharan Africa, rabies remains an endemic disease that is inadequately controlled. Studies have shown that economic constraints, limited access to health services, and a lack of awareness constitute major barriers to appropriate postexposure care [8, 15]. In this context, improving public knowledge and behaviors appears to be a key lever for reducing the incidence of the disease.

In the Republic of Guinea, rabies is a serious public health concern, characterized by a large number of stray dogs, low vaccination coverage, and limited public knowledge of the disease. Delayed access to care following exposure, often linked to economic and geographic constraints, increases the risk of human cases. However, available scientific data on the public’s KAP regarding rabies remain limited.

Rabies is endemic in Guinea but remains inadequately documented at the national level: Human cases continue to be reported sporadically, including in the capital, Conakry [16], and the country has not yet reached the World Health Organization’s “Zero by 30” target of eliminating dog‐mediated human rabies deaths by 2030 [17]. Existing control efforts rely mainly on opportunistic canine vaccination and PEP at a limited number of health facilities; to the authors’ knowledge, systematic mass dog‐vaccination campaigns, active surveillance, and a formal national elimination roadmap are not yet in place.

This lack of data constitutes an obstacle to the implementation of prevention strategies adapted to the local context. Indeed, a better understanding of the public’s knowledge, perceptions, and behaviors is necessary to effectively guide public health interventions and strengthen rabies control programs.

It is in this context that the present study was conducted. It aims to assess the KAP of the population regarding rabies in several regions of the Republic of Guinea, in order to identify existing gaps and contribute to the improvement of disease prevention and control strategies.

## 2. Materials and Methods

### 2.1. Study Area

The study was conducted in the Republic of Guinea, a West African country characterized by geographical, climatic, and sociocultural diversity. It took place in five administrative regions representative of the country, namely, Boké, Kindia, Labé, Siguiri, and N’Nzérékoré. The locations of the five study regions are shown in Figure 1.

**FIGURE 1:**
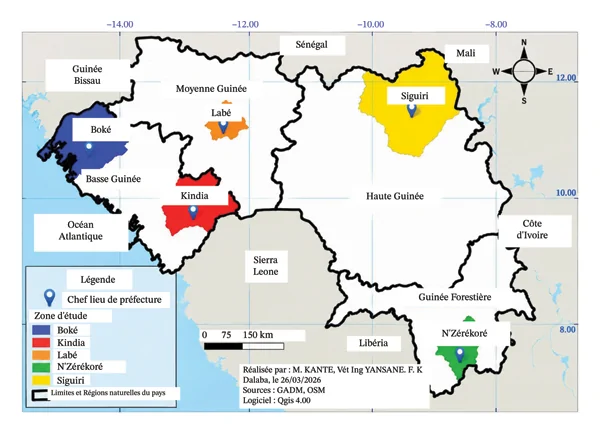
Map of the Republic of Guinea showing the location of the five study regions (Boké, Kindia, Labé, Siguiri, and N’Zérékoré).

These regions were selected to cover Guinea’s main ecological zones, namely, Maritime Guinea (Boké, Kindia), Middle Guinea (Labé), Upper Guinea (Siguiri), and Forest Guinea (N’Nzérékoré). This diversity allows for the consideration of environmental, socioeconomic, and cultural variations that may influence human–animal interactions, canine population dynamics, and practices related to rabies prevention.

The surveys were conducted primarily in urban and peri‐urban areas, where human and canine population densities are high, and where the risk of rabies transmission is potentially greater due to the frequent presence of stray dogs and variable access to veterinary and health services.

### 2.2. Sample Size

The minimum sample size was initially estimated using Schwartz’s formula:

(1) n=1.962×p1−pd2,

where *Z* = 1.96 (95% confidence level), *p* = 15% (expected prevalence, based on MIRAH Côte d’Ivoire data), and *d* = 0.05 (desired precision). This calculation yielded a minimum sample size of approximately 196 participants, representing the minimum number of respondents required under a simple random sampling design.

However, the present study was designed as a national cross‐sectional survey using a multistage cluster sampling strategy covering five administrative regions of the Republic of Guinea (Boké, Kindia, Labé, Siguiri, and N’Zérékoré). Therefore, the sample size obtained from Schwartz’s formula was considered only as the minimum statistical requirement. To ensure adequate geographical coverage of the five study regions, improve the representativeness of the target population, obtain sufficient observations for regional analyses, and address the operational constraints associated with a large multicenter survey, the operational sampling plan was expanded.

Because the final sample size was determined according to the operational national sampling plan rather than by applying a predefined numerical design effect, no specific design effect coefficient was used during sample size planning.

Accordingly, 3917 households were initially visited and screened across the five study regions, including 700 in Siguiri, 497 in Boké, 900 in Kindia, 911 in N’Zérékoré, and 909 in Labé (Table 1). One eligible respondent was identified in each household.

**TABLE 1:** Sociodemographic characteristics of respondents (*n* = 2733).

Variable	Category	Frequency (*n*)	Percentage (%)
Age group	18–34	1308	47.86
	35–49	971	35.53
	50+	454	16.61
	Total	2733	100.0

Education level	High school	902	33.0
	Not in school	799	29.24
	College	456	16.68
	Elementary	449	16.43
	Quranic education	127	4.65
	Total	2733	100.0

Occupation	Commerce/retail (retailer, tradespeople)	666	24.37
	Other (carpenters, masons, metalworkers, painters, welders, traders/vendors, and other low‐frequency skilled/informal occupations)	710	25.98
	Laborer/outdoor occupations (laborer, security guard, gold panner, hunter, Fisherman)	236	8.64
	Housewife	390	14.27
	Pupil/student (primary/secondary and university)	381	13.94
	Civil servant/health professions (civil servant, doctors, veterinarian)	205	7.50
	Agriculture/livestock (farmer, breeder)	145	5.31
	Total	2733	100.0

The number of households targeted in each region did not follow a strict population‐proportional allocation. According to 2014 General Population and Housing Census (RGPH)–based population estimates (Institut National de la Statistique, Perspectives démographiques de la Guinée), the total 2014 population was approximately 1,092,291 in Boké region, 1,573,690 in Kindia region, 1,001,392 in Labé region, 683,785 in Siguiri prefecture, and 1,591,716 in N’Zérékoré region [18]. A strict population‐proportional allocation of the 3917 households across these five areas (combined population 5,942,874) would therefore have yielded approximately 720 households in Boké, 1037 in Kindia, 660 in Labé, 451 in Siguiri, and 1049 in N’Zérékoré. The actual operational targets (497, 900, 909, 700, and 911, respectively) deliberately departed from this population‐proportional distribution: Labé and, in particular, Siguiri were allocated a larger‐than‐proportional share (909 vs. 660, and 700 vs. 451) to ensure adequate statistical power for regional‐level analyses despite their comparatively smaller population size, whereas Kindia and N’Zérékoré, despite larger populations, were allocated a smaller‐than‐proportional share (900 vs. 1,037, and 911 vs. 1049). Boké received the largest downward departure from its proportional share (497 vs. 720). This is attributable to the region’s ongoing bauxite‐mining boom, which has driven substantial in‐migration of mine workers and rapid, unplanned population growth in Boké; the resulting household instability and turnover complicated household listing and enumeration within the survey timeframe, limiting the number of households that could be reliably reached and screened relative to the population‐proportional target. This operational, capacity‐based allocation, rather than a purely population‐proportional one, explains the observed variation in household numbers across regions and is acknowledged, together with the absence of population‐proportional weighting, as a methodological limitation of the study.

Following the verification of questionnaire completeness and internal consistency during data quality control, 908 household records were excluded because they contained incomplete information or major inconsistencies. Consequently, 3009 household records with complete and internally consistent information were retained for participant recruitment.

Among the 3009 eligible individuals invited to participate, 276 declined participation after receiving detailed information about the study objectives and the verbal informed consent procedure. Therefore, 2733 participants voluntarily agreed to participate, completed the questionnaire, and were included in the final statistical analyses, corresponding to an overall participation rate of 90.83%.

The household and participant selection process is summarized in Figure 2.

**FIGURE 2:**
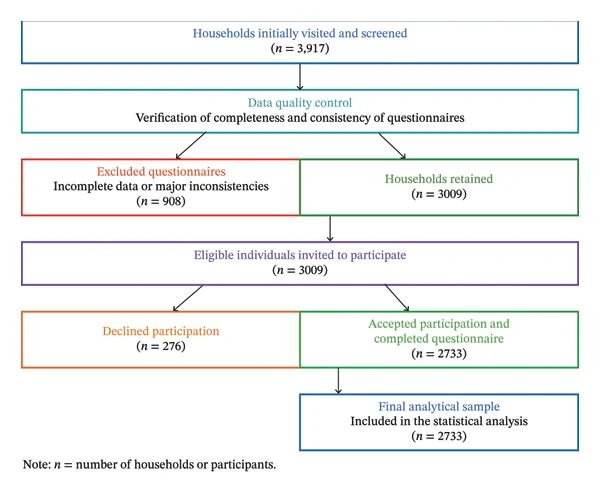
Flow diagram of the household and participant selection process. Note: *n* = number of households or participants.

### 2.3. Sampling

#### 2.3.1. National Sampling Frame

The sampling frame was derived from the 2014 RGPH, which provides the official administrative structure of the Republic of Guinea, including regions, prefectures, districts, and neighborhoods. This database served as the reference framework for selecting all sampling units included in the study.

A single multistage cluster sampling design was implemented throughout the study. The terms simple random sampling and systematic random sampling refer to the selection procedures applied at different stages of the same sampling design rather than to separate sampling methods.

During the first stage, the five study regions (Boké, Kindia, Labé, Siguiri, and N’Zérékoré) were purposively selected to ensure broad geographical coverage and adequate representation of Guinea’s major ecological zones.

During the second stage, eligible prefectures and neighborhoods within each selected region were selected by simple random sampling. Administrative units were first numbered according to the 2014 RGPH database, after which random numbers were generated using OpenEpi Version 3.01 to ensure a transparent, reproducible, and unbiased selection process.

During the third stage, households were selected by systematic random sampling within each selected neighborhood. A random starting point was chosen, and the sampling interval (*k*) was calculated by dividing the total number of households in each neighborhood by the target number of households to be surveyed. Households were then selected at regular intervals according to this sampling interval.

When more than one eligible adult was present in a selected household, the household head was interviewed whenever available. In the absence of the household head, another eligible adult household member (≥ 18 years) was selected according to the predefined field protocol.

Sampling weights were not applied because the primary objective of the study was to estimate overall KAP regarding rabies and to identify associated factors rather than to generate nationally weighted population estimates. Nevertheless, the absence of sampling weights and cluster‐adjusted variance estimation is acknowledged as a methodological limitation, as it may have resulted in a slight underestimation of standard errors.

#### 2.3.2. Sampling and Analysis Unit

The primary sampling unit was the household, whereas the unit of analysis was the individual respondent.

Only one eligible participant was interviewed in each selected household, thereby avoiding multiple observations from the same household and minimizing intrahousehold correlation.

### 2.4. Study Population

The study population consisted of adults residing in the selected households across the five study regions.

Although households constituted the primary sampling units, all KAP indicators and subsequent statistical analyses were performed at the individual level.

Eligible participants were adults aged 18 years or older who were present at the time of the survey, were able to understand and respond to the questionnaire, and voluntarily provided verbal informed consent before participation.

Only one respondent was included from each selected household.

Respondents were primarily heads of household or, in their absence, any other household member capable of providing reliable information. In some cases, a younger household member was interviewed when deemed capable of answering the questions.

This approach made it possible to collect data representative of KAP at the household level.

#### 2.4.1. Inclusion Criteria

Included in the study were individuals residing in the selected households who were present at the time of the survey and who gave free and informed consent. Respondents were primarily adults, or, failing that, any household member deemed capable of providing reliable information.

### 2.5. Data Collection Tool

Data were collected using a structured questionnaire specifically developed for this study based on internationally recognized rabies prevention guidelines, including those of Mission Rabies, the Global Alliance for Rabies Control (GARC), the WHO/OIE Action Plan for the Elimination of Dog‐Mediated Rabies, and relevant published literature on rabies KAP surveys. The questionnaire was specifically designed and adapted to the Guinean context to ensure its relevance to local epidemiological, sociocultural, and public health conditions.

The draft questionnaire underwent content validation by seven university faculty members with expertise in veterinary public health, epidemiology, infectious diseases, biostatistics, and One Health. Their recommendations were incorporated to improve the clarity, relevance, consistency, and scientific accuracy of the questionnaire before field implementation.

A pilot study was subsequently conducted in Dalaba Prefecture, which was not included among the study sites, to evaluate the clarity, comprehensibility, acceptability, and feasibility of the questionnaire under field conditions. Minor revisions were made following the pilot test before commencing data collection.

The final questionnaire comprised four sections:

Sociodemographic characteristics; knowledge about rabies (11 items); attitudes toward rabies prevention and control (4 items); and prevention and management practices (6 items).

The knowledge section assessed participants’ awareness of rabies, recognition of clinical signs in animals and humans, routes of transmission, animal reservoirs, disease fatality, availability of treatment after symptom onset, availability of human and animal vaccines, and recognized rabies control measures.

The attitude section assessed perceived responsibility for rabies prevention, willingness to pay for canine rabies vaccination, willingness to vaccinate dogs if vaccination were provided free of charge, and perceptions regarding the usefulness of rabies awareness campaigns.

The practice section assessed canine vaccination practices, actions taken following animal bites, observation of biting dogs, intended responses to suspected rabid animals, and previous participation in rabies awareness campaigns.

Correct or favorable responses were coded as 1, whereas incorrect, unfavorable, and “I don’t know” responses were coded as 0. For questions allowing multiple responses, scoring followed predefined criteria based on scientifically correct responses.

The internal consistency of the knowledge scale was assessed using Cronbach’s alpha coefficient and demonstrated good reliability (Cronbach’s *α* = 0.851). Because the attitude and practice sections comprised heterogeneous behavioral items, several of which were administered only to specific groups of respondents according to their characteristics (e.g., dog owners or respondents with previous exposure), these domains were not intended to measure a single latent construct. Therefore, Cronbach’s alpha was not considered an appropriate standalone indicator of reliability for the attitude and practice domains.

#### 2.5.1. Data Collection Procedure

Data were collected using the KoboCollect app, which enabled standardized digital data entry, reduced errors, and allowed for real‐time transmission.

#### 2.5.2. Operational Definition of Variables and Construction of KAP Scores

KAP variables were constructed based on responses to the structured questionnaire, using a standardized scoring approach inspired by the recommendations of the World Health Organization (WHO) and previous KAP studies on rabies.

##### 2.5.2.1. Knowledge

Knowledge levels were assessed using several questions regarding modes of transmission, prevention measures, and appropriate actions in case of exposure. Each correct answer was coded as 1, while incorrect answers or “I don’t know” responses were coded as 0. An overall score was obtained by summing the responses and then converted into a percentage of the maximum score.

Participants were classified into three categories:

• Good level of knowledge (≥ 70%),
• Average level (50%–69%),
• Low level (< 50%).

These thresholds, widely used in KAP studies, allow for the operational categorization of knowledge levels and facilitate comparability with previous work.

##### 2.5.2.2. Attitudes

Attitudes were assessed using items related to risk perception, the importance of vaccination, and behaviors regarding rabies. Favorable responses were coded as 1 and unfavorable responses as 0.

An overall attitude score was calculated and then converted to a percentage. An “acceptable attitude” was defined as a score ≥ 70%, a threshold commonly used in KAP studies to reflect a satisfactory level of adherence to recommended measures, while a score < 70% was considered unacceptable.

##### 2.5.2.3. Practices

Practices were assessed based on reported behaviors regarding prevention and postexposure management. Good practices were coded as 1 and poor practices as 0.

Scores were classified as follows:

 Good practices (≥ 70%),
 Average practices (50%–69%),
 Poor practices (< 50%).

This categorization is based on thresholds frequently used in KAP studies and allows for an operational interpretation of behaviors related to rabies prevention.

In addition, the internal consistency of the scores was assessed to verify the reliability of the scales used.

#### 2.5.3. Recruitment and Training of Interviewers

The surveys were conducted by five graduating veterinary medicine students who had previously been trained in questionnaire administration, interview techniques, data collection standardization, and ethical principles.

#### 2.5.4. Questionnaire Administration (KoboCollect)

The structured questionnaire was administered using the KoboCollect app, which enabled real‐time digital data collection. This tool facilitated the direct entry of responses, reduced transcription errors, and allowed for the geolocation of surveys. The questionnaire included sections on respondents’ sociodemographic characteristics, as well as their KAP regarding rabies.

#### 2.5.5. Questionnaire Pretest (Dalaba)

A pretest of the questionnaire was conducted in the city of Dalaba prior to the official launch of the survey. This pilot phase allowed for an assessment of the clarity, relevance, and participants’ understanding of the questions. Observations from the pretest led to adjustments to the questionnaire, including the reformulation of certain questions and improvements to the overall structure, in order to optimize the quality of the collected data.

#### 2.5.6. Interviews With Stakeholders (Regional Directorate of Veterinary Services [DRSV], Prefectural Directorates of Livestock [DPE], Municipalities)

Interviews were conducted with key institutional stakeholders, including the DRSV, the DPE, and municipal authorities (town halls). These discussions provided additional information on rabies management, implemented prevention strategies, and local constraints. They also facilitated access to communities and the logistical organization of the surveys.

#### 2.5.7. Household Survey

The survey was conducted among selected households in the study areas. Interviews were conducted face‐to‐face using a structured questionnaire administered by pretrained interviewers.

Participants were primarily heads of households, due to their central role in managing household information. In their absence, any person residing in the household who was able to provide reliable information was interviewed. In some cases, a younger household member was included when deemed capable of answering the questions correctly.

Data were collected anonymously and confidentially, in accordance with current ethical principles. The use of the KoboCollect app enabled direct digital entry of responses, reducing transcription errors and facilitating real‐time data management.

### 2.6. Data Analysis

The collected data were exported, verified, cleaned, coded, and analyzed using DATAtab (Version 1.0) and R statistical software (Version 4.4.1; R Foundation for Statistical Computing, Vienna, Austria). Descriptive statistics were used to summarize the characteristics of the study population. Categorical variables were presented as frequencies and percentages. Associations between categorical variables were initially assessed using Pearson’s chi‐square test. Fisher’s exact test was applied whenever expected cell frequencies were less than five.

To identify factors associated with the study outcomes, two separate multivariable binary logistic regression models were fitted. The first model evaluated factors associated with acceptable attitude toward rabies prevention (acceptable vs. unacceptable attitude), while the second model evaluated factors associated with dog‐bite occurrence (yes vs. no). Knowledge and practice scores were analyzed descriptively and were not modeled using logistic regression. Throughout this manuscript, the term “multivariable analysis” is used consistently to refer to multivariable binary logistic regression, in which a single binary outcome variable is analyzed simultaneously with several explanatory variables.

Variables showing evidence of association in the univariable analyses (*P* < 0.20) or considered epidemiologically relevant based on previous literature were considered candidates for inclusion in the multivariable models. Adjusted odds ratios (aORs) with their corresponding 95% confidence intervals (95% CIs) were reported. Multicollinearity among explanatory variables was assessed using the variance inflation factor (VIF). Variables with VIF values below 5 were considered to show no evidence of problematic multicollinearity. All statistical tests were two‐sided, and statistical significance was established at *p* < 0.05. Probability values were reported according to standard scientific conventions, with *p* < 0.001 reported where appropriate.

## 3. Results

The survey results regarding the KAP of respondents in Guinea regarding rabies. The analysis highlights the level of understanding of the disease, perceptions associated with the risk of transmission, as well as the prevention and management behaviors adopted by the population.

A total of 2733 participants were included in the study. For descriptive purposes, age was further grouped into three broader categories. The age distribution was relatively balanced across these categories, with 18–34 years representing the largest group (1308; 47.86%), followed by 35–49 years (971; 35.53%) and 50 years and older (454; 16.61%), indicating that respondents covered a broad range of adult age categories.

Regarding educational attainment, high school education was the most common level (902; 33.0%), followed by participants with no formal schooling (799; 29.24%). College education (456; 16.68%) and elementary education (449; 16.43%) were similarly represented, whereas Quranic education accounted for the smallest proportion (127; 4.65%).

For descriptive purposes, the numerous low‐frequency individual occupations (each accounting for less than 1.6% of respondents) were regrouped into fewer, more representative categories, while occupations already representing a distinct and sizeable group were kept separate. The largest category remained other (710; 25.98%), comprising various low‐frequency informal or skilled occupations such as carpenters, masons, metalworkers, painters, welders, and traders/vendors, none of which could be individually identified with sufficient precision in the dataset to form standalone categories. Commerce/retail (retailers and tradespeople combined) was the second largest group (666; 24.37%), followed by housewives (390; 14.27%), pupils/students (primary, secondary, and university combined; 381; 13.94%), laborers and outdoor occupations (laborers, security guards, gold panners, hunters, and fishermen combined; 236; 8.64%), civil servants and health professions (civil servants, doctors, and veterinarians combined; 205; 7.50%), and agriculture/livestock (farmers and breeders combined; 145; 5.31%).

### 3.1. Public Knowledge of Rabies Transmission

To assess the population’s level of knowledge regarding the modes of rabies transmission, a survey was conducted. These results are presented in Table 2.

**TABLE 2:** Knowledge of rabies transmission routes among respondents (*n* = 2733).

Transmission routes	Frequency (*n*)	Percentage (%)
Bites	1035	37.87
Scratches	106	3.88
Licking a wound	46	1.68
Affectionate_contact	45	1.65
Consumption_of_dog_by‐products	102	3.73

Biting was identified as the primary route of rabies transmission (37.87%), followed by scratching (3.88%), consumption of dog by‐products (3.73%), licking of wounds (1.68%), and affectionate contact with dogs (1.65%). A single respondent (0.04%) identified witchcraft as a route of rabies transmission, whereas no participant (0%) reported insect bites as a transmission route.

### 3.2. Knowledge of Rabies Prevention Measures Among Respondents

The results highlight the population’s knowledge of rabies prevention measures, allowing for the identification of recognized practices as well as potential gaps in prevention. These results are presented in Table 3.

**TABLE 3:** Knowledge of rabies prevention measures among respondents (*n* = 2733).

Preventive measures	Frequencies (*n*)	Percentage (%)
Getting vaccinated	248	9.07
Have your dog vaccinated	1230	45.01
Controlling the dog population	144	5.27
Euthanize all stray dogs	205	7.5
Care for people already exposed	39	1.43
Prevention campaign	356	13.03

Based on the results in this table, dog vaccination was identified as the primary prevention measure (45.01%), followed by awareness campaigns (13.03%) and human vaccination (9.07%). Other measures cited include the culling of stray dogs (7.5%) and canine population control (5.27%).

### 3.3. Factors Associated With an Acceptable Attitude Toward Rabies Prevention (*N* = 2733)

To identify the determinants of attitudes toward rabies prevention, a multivariable logistic regression analysis was conducted. This approach allowed for the evaluation of the association between respondents’ sociodemographic characteristics and the probability of adopting an acceptable attitude. The results of this analysis are presented in Table 4.

**TABLE 4:** Factors associated with an acceptable attitude toward rabies prevention (*n* = 2733).

Variable	Adjusted OR	95% CI	*p* value
Primary school vs. no schooling	1.28	1.05–1.56	0.020
Secondary school vs. no schooling	1.64	1.32–2.03	< 0.001
Higher education vs. no schooling	2.45	1.50–3.98	< 0.001
25–34 years vs. 18–24 years	1.31	1.08–1.59	0.006
35–49 years vs. 18–24 years	1.47	1.20–1.80	< 0.001
≥ 50 years vs. 18–24 years	1.22	0.98–1.51	0.070
Retailer vs. other	1.36	1.12–1.65	0.002
Civil servant vs. other	1.82	1.20–2.75	0.004
Student^∗^ vs. other	1.55	1.10–2.18	0.010
Farmer vs. other	0.88	0.65–1.19	0.400

^∗^For the multivariable logistic regression, pupils (primary/secondary school) and university students were analyzed as a single “Student” category because this was the coding used in the regression model. For descriptive purposes (Table 1), this category was subsequently separated into “pupil (primary/secondary school)” and “university student” to provide a more detailed description of the study population.

Multivariable logistic regression identified educational level, selected age groups, and occupation as factors significantly associated with an acceptable attitude toward rabies prevention.

### 3.4. Respondents’ Practices Regarding Rabies

This table presents respondents’ practices regarding rabies, including case management behaviors as well as preventive measures such as vaccination and awareness‐raising. Respondents’ practices regarding rabies are presented in Table 5.

**TABLE 5:** Respondents’ practices regarding rabies (*n* = 2733).

Domain	Indicator	Yes *n* (%)	No *n* (%)
Perceptions (misconceptions)	Believes that treatment is possible after symptoms appear in men	642 (23.49)	2091 (76.51)
	Believes that treatment is possible after symptoms appear in animals	570 (20.86)	2163 (79.14)

Prevention practices	Dog vaccination	1585 (58.0)	1148 (42.0)
	Postexposure prophylaxis (PEP) for humans	1450 (53.0)	1283 (47.0)
	Participation in awareness campaigns	1200 (43.9)	1533 (56.1)

The results show significant gaps in the understanding of rabies among the study population. In fact, nearly a quarter of respondents (23.49% for humans and 20.86% for animals) mistakenly believe that treatment is possible after symptoms appear, reflecting a critical lack of awareness regarding the almost invariably fatal nature of clinical rabies.

On the other hand, certain preventive practices are relatively better known and adopted, notably dog vaccination (58.0%) and the use of postexposure human vaccination (53.0%). However, low participation in awareness campaigns (43.9%) suggests that public health messages have not yet been sufficiently disseminated.

Overall, these results highlight a disconnect between certain preventive practices and the actual level of knowledge, which could limit the effectiveness of rabies control strategies in Guinea.

Distribution of KAP scores.

Table 6 summarizes the distribution of participants according to their KAP regarding rabies.

**TABLE 6:** Distribution of knowledge, attitudes, and practices (KAP) scores.

Level	Knowledge *n* (%)	Attitudes *n* (%)	Practices *n* (%)
Low/poor	1450 (53.10)	2428 (88.84)	2017 (73.80)
Average/moderate	800 (29.30)	295 (10.79)	220 (8.05)
Good	483 (17.60)	10 (0.37)	496 (18.15)
Total	**2733 (100.0)**	**2733 (100.0)**	**2733 (100)**

*Note:* The bold values in the total row represent the overall number of respondents (*n* = 2733) and the corresponding percentage (100%) for each KAP domain.

The distribution of KAP scores showed that more than half of the participants had a low level of knowledge about rabies (1450/2733; 53.10%), whereas 29.30% (800/2733) demonstrated an average level of knowledge and only 17.60% (483/2733) had good knowledge. Attitudes toward rabies prevention were predominantly unfavorable, with 88.84% (2428/2733) of respondents classified as having low attitude scores, while 10.79% (295/2733) and only 0.37% (10/2733) exhibited moderate and good attitudes, respectively. Similarly, preventive practices were generally inadequate, as 73.80% (2017/2733) of participants had poor practices. In contrast, 8.05% (220/2733) demonstrated average practices, whereas only 18.15% (496/2733) exhibited good practices. Overall, the findings indicate low levels of knowledge, attitudes, and preventive practices regarding rabies among the study population.

### 3.5. Factors Associated With Dog Bites by Age and Educational Level

The table presents the results of the analysis of factors associated with dog bites based on the participants’ age group and educational level. The corresponding crude and adjusted associations are presented in Table 7.

**TABLE 7:** Factors associated with dog bites by age and education level.

Variables	Categories	Crude OR (95% CI)	*p* value	Adjusted OR (95% CI)	*p* value
Age	18–24 (ref.)	1	—	1	—
	25–29	1.35 (1.05–1.74)	0.02	1.28 (0.98–1.67)	0.07
	30–34	1.31 (1.01–1.70)	0.04	1.22 (0.93–1.60)	0.14
	35–39	1.34 (1.02–1.76)	0.03	1.26 (0.95–1.67)	0.10
	40–44	1.27 (0.96–1.67)	0.09	1.18 (0.88–1.57)	0.26
	45–49	1.24 (0.91–1.70)	0.17	1.15 (0.83–1.59)	0.40
	≥ 50	1.45 (1.11–1.89)	0.006	1.98 (1.20–3.25)	0.006

Level of education	Quranic (ref.)	1	—	1	—
	Not in school	0.88 (0.58–1.32)	0.53	0.94 (0.61–1.44)	0.78
	Elementary	0.98 (0.63–1.53)	0.93	1.05 (0.66–1.67)	0.83
	Secondary	0.53 (0.30–0.92)	0.025	0.62 (0.34–1.12)	0.10
	College	0.57 (0.31–1.05)	0.07	0.69 (0.36–1.32)	0.27

The bivariate analysis shows that the age groups 25–29, 30–34, and 35–39 are associated with an increased risk of dog bites (*p* < 0.05). After adjustment, these associations are no longer statistically significant. In contrast, individuals aged 50 and older have a significantly higher risk of being bitten, both in the crude analysis (OR = 1.45; 95% CI: 1.11–1.89; *p* = 0.006) and in the multivariable analysis (ORa = 1.98; 95% CI: 1.20–3.25; *p* = 0.006).

Regarding educational level, no significant association was observed in the multivariable analysis. However, in the bivariate analysis, a high school education was associated with a reduced risk of being bitten (OR = 0.53; 95% CI: 0.30–0.92; *p* = 0.025).

### 3.6. Factors Associated With Dog Bites According to Dog Ownership, Vaccination Status, and Dogs’ Living Conditions

The table presents the results of the analysis of factors associated with dog bites based on characteristics related to dog ownership, dog management, and the level of knowledge about rabies in the study population. Binary logistic regression was used to estimate crude and aORs, along with their 95% CIs. Reference categories were defined for each variable to allow for a meaningful comparison between groups. The results of these analyses are presented in Table 8.

**TABLE 8:** Factors associated with dog bites according to dog ownership, vaccination status, and dogs’ living conditions (*n* = 2733).

Variables	Categories	Population	Adjusted OR	95% CI	*p* value
Dog ownership	Yes vs. no	Overall (*n* = 2733)	0.99	(0.82–1.20)	0.90
Knowledge of rabies	Yes vs. no	Overall (*n* = 2733)	0.94	(0.80–1.10)	0.45
Dog‐vaccination status	Yes vs. no	Owners (*n* = 399)	1.10	(0.74–1.63)	0.63
Dog’s lifestyle (free‐roaming)	Yes vs. no	Owners (*n* = 399)	0.75	(0.50–1.14)	0.18
Knowledge of rabies	Yes vs. no	Owners (*n* = 399)	0.93	(0.63–1.37)	0.72

Multivariable analysis did not identify any factors significantly associated with dog bites among variables related to dog ownership, vaccination status, and dog lifestyle. Only age was significantly associated with dog bites in the overall analysis.

Dog ownership (ORa = 0.99; 95% CI: 0.82–1.20; *p* = 0.90) and knowledge of rabies (ORa = 0.94; 95% CI: 0.80–1.10; *p* = 0.45) were not associated with the risk of being bitten. Among dog owners, neither vaccination status (ORa = 1.10; 95% CI: 0.74–1.63; *p* = 0.63) nor the dog’s lifestyle (ORa = 0.75; 95% CI: 0.50–1.14; *p* = 0.18), nor level of knowledge (ORa = 0.93; 95% CI: 0.63–1.37; *p* = 0.72) were significantly associated with bites.

### 3.7. Relationship Between Knowledge of Rabies and Postexposure Practices

The table presents the relationship between knowledge of rabies and postexposure practices among individuals who had been bitten by a dog. A cross‐tabulation analysis was conducted to assess the influence of knowledge level on the adoption of appropriate behaviors following exposure. The chi‐square test was used to determine the existence of a statistically significant association between these variables. The cross‐tabulation results are presented in Table 9.

**TABLE 9:** Relationship between knowledge of rabies and postexposure practices (*n* = 987).

Knowledge of rabies	I don’t know *n* (%)	No *n* (%)	Yes *n* (%)	Total	*p* value
Yes	313 (48.8%)	113 (17.6%)	216 (33.6%)	642	
No	320 (92.8%)	17 (4.9%)	8 (2.3%)	345	
Total	633	130	224	987	< 0.001

A significant association was observed between knowledge of rabies and postexposure practices (*p* < 0.001). Among respondents who had heard of rabies, 33.6% took appropriate action, 48.8% did not know what to do, and 17.6% took no action. Among respondents who were unaware of rabies, 92.8% did not know what to do, while 2.3% sought medical care.

### 3.8. Regional Distribution of Key Knowledge, Prevention Awareness, and Outcome Indicators (*N* = 2733)

Table 10 presents the regional distribution of selected rabies‐related knowledge, awareness, and exposure indicators across the five study regions of Guinea (Boké, Kindia, Labé, Siguiri, and N’Zérékoré). Significant regional differences were observed for all knowledge and prevention indicators (*p* < 0.001), whereas the proportion of respondents reporting a previous dog bite did not differ significantly between regions (*p* = 0.845).

**TABLE 10:** Regional distribution of key knowledge, prevention awareness, and outcome indicators (*n* = 2733).

Indicator	Boké (*n* = 453)	Kindia (*n* = 462)	Labé (*n* = 573)	Siguiri (*n* = 535)	N’Zérékoré (*n* = 710)	*p* value^∗^
Heard of rabies (general awareness), %	62.7^b^	43.7^a^	50.8^a^	83.7^c^	81.4^c^	< 0.001
Correctly identified bites as the main transmission route, %	35.3^ab^	40.9^bc^	36.1^ab^	28.4^a^	46.1^c^	< 0.001
Cited dog vaccination as a prevention measure, %	30.7^b^	26.8^b^	14.0^a^	84.3^d^	61.4^c^	< 0.001
Misconception: believes treatment is possible after human symptoms appear, %	34.7^d^	5.2^a^	10.3^b^	26.2^c^	36.9^d^	< 0.001
Ever bitten by a dog, %	34.9	35.7	37.7	35.0	36.8	0.845

*Note:* For each indicator showing a significant overall regional association (*p* < 0.001), pairwise comparisons between regions were subsequently performed using two‐proportion *z*‐tests with Bonferroni correction for multiple comparisons (10 pairwise tests per indicator; adjusted significance threshold *p* < 0.005). Within each indicator, regions sharing the same superscript letter (a, b, c, d) did not differ significantly from one another, whereas regions with different letters differed significantly.

^∗^Pearson’s chi‐square test, 4 degrees of freedom.

General awareness of rabies, correct knowledge of dog bites as the main transmission route, recognition of dog vaccination as a prevention measure, and the misconception that treatment remains possible after symptom onset all varied significantly by region (*p* < 0.001 for all four indicators). Awareness and knowledge of dog vaccination were highest in Siguiri and N’Zérékoré and lowest in Kindia and Labé, while the treatment misconception was most prevalent in Boké and N’Zérékoré and least prevalent in Kindia. In contrast, the proportion of respondents ever bitten by a dog did not differ significantly across regions (34.9%–37.7%; *p* = 0.845), suggesting that regional variation in knowledge does not translate into a detectable difference in bite risk, consistent with the multifactorial determinants of dog bites discussed below.

## 4. Discussion

### 4.1. Sociodemographic Characteristics

The results show that the study population is relatively evenly distributed across different age groups, with a slight predominance of individuals aged 50 and older (16.61%), followed by the 25–29 age group (16.47%) and the 18–24 age group (16.06%). This distribution is a methodological strength, as it allows for the inclusion of both young adults and older individuals, thereby providing better representativeness for the analysis of KAP regarding rabies. In KAP surveys, age is recognized as an important determinant influencing exposure to dogs and healthcare‐seeking behaviors [3, 19].

The educational level is dominated by secondary education (33.0%), followed by those with no formal education (29.24%). This distribution is particularly relevant, as education is generally associated with a better understanding of zoonotic diseases and the adoption of preventive behaviors. However, the high proportion of people with no formal education poses a challenge to the effective dissemination of public health messages, particularly in areas with limited access to information [10, 14].

The distribution of occupations, marked by a predominance of the informal sector (traders and other activities), reflects the socioeconomic context of the areas studied in Guinea, notably in Boké, Kindia, Labé, Siguiri, and N’Zérékoré. These contexts influence human–animal interactions and exposure to dogs, particularly in urban and peri‐urban areas where stray dogs are common [7].

### 4.2. Awareness of Rabies

In this study, 66% of respondents reported having heard of rabies, while 34% had no knowledge of it, indicating an overall level of awareness that remains insufficient.

Although a majority of respondents were aware of rabies, this level of awareness remains insufficient given that rabies is an almost invariably fatal yet entirely preventable disease. Incomplete awareness may reduce timely health‐seeking behavior following exposure and limit participation in canine vaccination programs. These findings highlight the need to strengthen community‐based rabies education and awareness strategies, particularly through locally accessible communication channels, as part of an integrated One Health approach to rabies prevention and control.

These results are comparable to those observed in several endemic countries. In Tanzania, Sambo et al. [10] reported similar levels of knowledge, although these varied by region. Similarly, in Ethiopia, Deressa et al. [9] highlighted significant gaps in understanding of the disease. Globally, Hampson et al. [3] emphasize that, despite some awareness of rabies, knowledge often remains superficial in resource‐limited countries. Furthermore, in Asia, Acharya et al. [14] also reported that awareness of rabies does not guarantee an adequate understanding of the risks.

### 4.3. Modes of Transmission

In this study, bites were identified as the primary mode of transmission by 37.87% of respondents, which remains relatively low compared to established scientific knowledge, according to which more than 99% of human rabies cases are linked to dog bites. Other reported modes of transmission include scratches (3.88%) and licking of wounds (1.68%), while some responses reflect misconceptions, notably affectionate contact (1.65%) or witchcraft (0.04%). These results are comparable to those observed in several endemic settings. In Tanzania, Sambo et al. [10] showed that, although bites are generally identified as the primary mode of transmission, a significant proportion of the population has incomplete knowledge of transmission mechanisms. Similarly, in Ethiopia, Deressa et al. [9] reported that populations recognize bites but also hold misconceptions about other modes of transmission. In West Africa, Tschopp et al. [20] also highlighted the persistence of sociocultural beliefs influencing perceptions of the disease.

Internationally, Hemachudha et al. [21] confirm that, although modes of transmission other than bites are biologically possible, their contribution remains marginal. Furthermore, the World Health Organization [4] emphasizes that bites are the almost exclusive route of rabies transmission in humans.

### 4.4. Preventive Measures Against Rabies

In this study, 45.01% of respondents identified dog vaccination as the primary preventive measure, followed by awareness campaigns (13.03%) and human vaccination (9.07%). However, inappropriate strategies such as the culling of stray dogs (7.5%) were also mentioned, reflecting a limited understanding of control measures.

These findings are comparable to those observed in several endemic countries. In Tanzania, Cleaveland et al. [7] showed that canine vaccination is widely recognized but remains poorly understood as a key elimination strategy. Similarly, in Ethiopia, Deressa et al. [9] reported limited knowledge of comprehensive prevention measures, particularly regarding postexposure management. In Asia, Acharya et al. [14] also observed that populations have only partial awareness of preventive measures, with limited knowledge of what to do after exposure.

Globally, Hampson et al. [3] emphasize that dog vaccination is the most effective strategy for interrupting rabies transmission, provided that the coverage of at least 70% is achieved. Furthermore, the WHO [4] stresses the importance of an integrated approach combining animal vaccination, human care, and community awareness.

Overall, these results indicate that, although certain key measures are known, understanding of them remains incomplete, which may limit the effectiveness of prevention strategies.

The results show that 73.80% of respondents exhibit poor practices, compared to 18.15% with good practices and 8.05% with average practices, reflecting a significant gap between knowledge and actual behavior.

In particular, a significant proportion of respondents (23.49% among humans and 20.86% among animals) hold the mistaken belief that treatment is possible after symptoms appear, whereas rabies is almost always fatal at this stage. This confusion constitutes a major obstacle to seeking early care and using PEP.

These results are similar to those observed in several studies. In Africa, Knobel et al. [15] demonstrated that economic constraints and limited access to care strongly influence postexposure behaviors. Zinsstag et al. [22] also highlighted that structural factors constitute major barriers to adequate care. In Ethiopia, Deressa et al. [9] reported that, despite some knowledge of rabies, practices remain inadequate. Similarly, in Tanzania, Sambo et al. [10] highlighted a persistent disconnect between knowledge and behavior.

Internationally, these findings confirm that improving practices requires not only better information but also enhanced access to health services and interventions tailored to the sociocultural context.

### 4.5. Factors Associated With Dog Bites

The multivariable analysis did not identify any factors significantly associated with dog bites among the variables related to dog ownership, vaccination status, dog living conditions, and level of knowledge. However, age ≥ 50 years was identified as a significantly associated factor in the overall analysis.

The lack of a significant association between most of the factors examined and dog bites suggests that these events may be influenced by complex environmental, behavioral, and structural determinants that were not fully captured in the present study.

The lack of an effect of vaccination status is consistent, as vaccination prevents virus transmission but does not influence aggressive behavior in dogs. Similarly, knowledge of rabies is not associated with bites, confirming the dissociation between knowledge and behavior [23]. A comparably large KAP and dog‐ecology survey in Namibia similarly found no significant association between dog ownership/vaccination status and human dog‐bite risk [24].

Bites thus appear to be more closely linked to

• unpredictable human–animal interactions
• the presence of stray dogs
• environmental factors
• risky human behaviors.

These findings are consistent with the work of Messam et al. [25] and Gilchrist et al. [26].

### 4.6. Relationship Between Knowledge and Postexposure Practices

A statistically significant association was observed between knowledge of rabies and postexposure practices (*p* < 0.001). Among informed individuals, 33.6% adopt appropriate behavior, while 48.8% report not knowing what to do, and 7.6% adopt inappropriate practices.

In contrast, among uninformed individuals, 92.8% reported not knowing what to do, highlighting the major influence of knowledge levels on behavior.

These results are comparable to those reported in several studies. In Ethiopia, Deressa et al. [9] showed that knowledge of rabies improves practices, although it does not guarantee appropriate behaviors. Similarly, in Tanzania, Sambo et al. [10] highlighted significant gaps in postexposure practices, even among informed individuals.

These results confirm that, although knowledge is an important determinant of behavior, it must be accompanied by targeted, action‐oriented communication strategies to promote the adoption of appropriate practices.

They also highlight that a lack of knowledge is a key factor in risky behaviors and that public health interventions must prioritize improving knowledge in order to bring about lasting changes in practices.

### 4.7. Ethics Statement

This study received ethical approval from the Ethics and Deontology Committee (CED) of the Higher Institute of Veterinary Sciences and Medicine of Dalaba (ISSMV/D), through its General Directorate of Research (Authorization Reference No. 2025/640/ISSMV/DGR/CED). All procedures were conducted in accordance with applicable institutional guidelines and regulations and with the principles of the Declaration of Helsinki. Prior to inclusion, all participants were informed of the study’s objectives and procedures. Free, informed, and verbal consent was obtained from each participant; because the majority of respondents were illiterate, verbal consent was explicitly authorized by the ethics committee in place of written consent. Data were collected and analyzed anonymously and confidentially.

## 5. Study Limitations

Several operational constraints were encountered in the field, including difficulties accessing certain areas, language barriers, varying levels of understanding of the questions by respondents, and the potential influence of interviewers on responses. Additionally, the unavailability of certain household heads and logistical constraints may have affected data collection.

In addition, this study has several methodological limitations. First, the cross‐sectional design precludes causal inference regarding the observed associations, including those identified in the attitude and dog‐bite models. Second, the KAP data were self‐reported and may therefore be affected by recall bias and social desirability bias, potentially leading to an overestimation of favorable practices. Third, although data collectors received standardized training, data collection was conducted by veterinary students rather than the investigators, introducing the possibility of interviewer bias. Fourth, the multivariable analyses included only a limited number of explanatory variables, which may have restricted the identification of additional determinants. Finally, although the survey covered five ecologically distinct regions of Guinea, the regional comparison reported in the Results section (Table 10) relies on individual indicator variables rather than the composite KAP scores themselves, since the latter could not be independently rederived from the exported dataset with full certainty; a region‐stratified analysis of the composite KAP scores using the original scoring syntax remains a priority for future analysis of this dataset.

Future research should include longitudinal or incidence‐based epidemiological studies to establish temporal and causal relationships.

## 6. Conclusion

This study highlights that rabies remains a serious public health concern in the Republic of Guinea, characterized by a marked disconnect between the level of knowledge and the behaviors adopted by the population. Despite a relatively high proportion of people having heard of the disease, knowledge remains incomplete and sometimes erroneous, particularly regarding modes of transmission and effective prevention measures.

The results reveal low adherence to recommended attitudes and practices, with a predominance of inappropriate behaviors, particularly in postexposure management. This situation exposes populations to an increased risk of transmission, in a context where the availability and accessibility of health and veterinary services remain limited.

The lack of a significant association between most of the factors examined and dog bites suggests that these events may be influenced by complex environmental, behavioral, and structural determinants that were not fully captured in the present study.

Based on these human‐focused findings, future rabies control strategies should consider integrating multisectoral approaches within a One Health framework.

A coordinated approach involving the human, animal, and environmental health sectors is essential to sustainably reduce the burden of rabies in Guinea.

## Author Contributions

Noumouké Kante: conceptualization, methodology, formal analysis, fieldwork, and first draft; Abdoulaye Kuan Traore: supervision and validation; Lanceï Kaba: conceptualization and statistical data analysis; Almamy Ousmane Deen Camara and Yacouba Konate: protocol and manuscript revision; Cathérine Beimy, Pathé Navabb Kowane, Pépé Koly Guilavogui, Michel Alexis Sagno, and Moussa Camara: the survey and field data collection; Karim Tuo and Madou Sanou: manuscript revision; Mohamed Magassouba, Kouramoudou Berete, Mohamed Idriss Doumbouya, Pépé Gbamou, Sayon Yombouno, Fassou Kourouma, and Nafissatou Ouedraogo: methodology, research, and visualization; Noël Tordo: expert validation of scientific content and review of virological aspects; Nicolas Barro: supervision and final approval of the manuscript prior to submission. Noumouké Kante, the corresponding author, had full access to all of the data in this study and takes full responsibility for the integrity of the data and the accuracy of the data analysis.

Noumouké Kante is the manuscript guarantor and, as the corresponding author, had full access to all of the data in this study and takes complete responsibility for the integrity of the data and the accuracy of the data analysis.

## Funding

This study received no specific funding.

## Disclosure

Noumouké Kante, as the manuscript guarantor, affirms that this manuscript is an honest, accurate, and transparent account of the study being reported, that no important aspects of the study have been omitted, and that any discrepancies from the study as originally planned have been explained. No funding source had any role in the study design, data collection, data analysis, data interpretation, manuscript preparation, or the decision to submit the manuscript for publication.

## Conflicts of Interest

The authors declare no conflicts of interest.

## Data Availability

The datasets are available from the corresponding author upon reasonable request.
